# Nitrative Stress-Related Autophagic Insufficiency Participates in Hyperhomocysteinemia-Induced Renal Aging

**DOI:** 10.1155/2020/4252047

**Published:** 2020-01-25

**Authors:** Shangyue Zhang, Yuerong Zhang, Xinyu Zhang, Chenghua Luo, Yan Cao, Dengyu Ji, Wenjing Yan, Ke Xue, Jiayin Chai, Hongyan Dai, Wen Wang

**Affiliations:** ^1^Department of Physiology and Pathophysiology, School of Basic Medical Sciences, Capital Medical University, Beijing, China; ^2^Department of Cardiology, Qingdao Municipal Hospital, Qingdao, Shandong, China; ^3^Beijing Key Laboratory of Metabolic Disorders Related Cardiovascular Diseases, Capital Medical University, Beijing, China

## Abstract

The kidneys are important organs that are susceptible to aging. Hyperhomocysteinemia (HHcy) is a risk factor for nephropathy and is associated with chronic nephritis, purpuric nephritis, and nephrotic syndrome. Numerous studies have shown that elevated serum homocysteine levels can damage the kidneys; however, the underlying mechanism of HHcy on kidney damage remains unclear. In this study, we make use of a diet-induced HHcy rat model and in vitro cell culture to explore the role of autophagy in HHcy-induced renal aging and further explored the underlying mechanism. We demonstrated that HHcy led to the development of renal aging. Promoted kidney aging and autophagic insufficiency were involved in HHcy-induced renal aging. HHcy decreased the expression of transcription factor EB (TFEB), the key transcription factor of autophagy-related genes in renal tissue. Further experiments showed that nitrative stress levels were increased in the kidney of HHcy rats. Interestingly, pretreatment with the peroxynitrite (ONOO^−^) scavenger FeTMPyP not only reduced the Hcy-induced nitrative stress in vitro but also partially attenuated the decrease in TFEB in both protein and mRNA levels. Moreover, our results indicated that HHcy reduced TFEB expression and inhibited TFEB-mediated autophagy activation by elevating nitrative stress. In conclusion, this study showed an important role of autophagic insufficiency in HHcy-induced renal aging, in which downregulation of TFEB plays a major role. Furthermore, downexpression of TFEB was associated with increased nitrative stress in HHcy. This study provides a novel insight into the mechanism and therapeutic strategy for renal aging.

## 1. Introduction

The incidence of kidney diseases gradually increases in the elderly. A thorough understanding of the mechanisms involved in kidney aging is of great significance for the prevention of kidney disease. As a sulfur-containing amino acid formed by demethylation of methionine in the body, homocysteine (Hcy) participates in important physiological processes such as energy metabolism and methylation. Kidneys are key organs for the metabolism of Hcy, and the accumulation of Hcy in serum is closely related to kidney diseases. Hyperhomocysteinemia (HHcy) is associated with kidney damage [1]. Studies have shown that there are various degrees of Hcy metabolic abnormalities in various kidney diseases [2, 3], such as nephrotic syndrome [4], lupus nephritis [5], and chronic nephritis [3, 6, 7], most of which are accompanied by HHcy. On the other hand, HHcy also shows an influence on the kidney, causing changes in kidney structure and function and exacerbating kidney damage. In recent years, scholars have reached a common consensus that HHcy is an important independent risk factor for kidney disease. However, whether or not HHcy can directly induce the progress of renal aging still remains unknown.

HHcy-induced kidney damage may be associated with multiple factors such as increased oxidative stress [8] and endoplasmic reticulum stress [9], inhibition of DNA methylation [10], and homocysteine modification of protein [11]. Under physiological conditions, cells display a universal self-protective mechanism, namely, autophagy. Autophagy is a process in which eukaryotic cells regulate degradation of damaged organelles and proteins. It plays a key role in maintaining a cell dynamic equilibrium steady state [12]. Recent studies have found that activation of autophagy protects the kidney congenital cells and reduces kidney injury, such as renal interstitial fibrosis and necrosis of renal tubular epithelial cells [13, 14]. Podocytes are an important component of the glomerular filtration barrier. They are terminally differentiated cells without division and proliferation. Immune damage and oxidative stress can cause podocyte damage, dysfunction, and even apoptosis. Podocyte injury can lead to a variety of diseases, and autophagy has been shown to be important for the growth and development of these cells [15–17].

In this study, we aimed to explore the role of HHcy in renal aging and demonstrate a specific regulatory mechanism of autophagy abnormality induced by HHcy.

## 2. Material and Methods

### 2.1. Ethics and Clinical Experiment

The serum samples were collected from patients with coronary heart disease (CHD) at the Department of Cardiology of Xuanwu Hospital, Capital Medical University. Exclusion criteria are patients younger than 18 years old and having a previous history of malignant tumors, diabetes, autoimmune diseases, and infectious diseases. All subjects were given written informed consent after being informed of the purpose and nature of the study. All serum samples were collected after 8 hours of fasting and the renal function test, and Hcy levels were measured with an automated biochemical analyzer (Hitachi 7020, Tokyo, Japan) in a hospital clinical laboratory. Beclin-1 levels in serum of CHD patients were measured using a commercial ELISA kit (TB Healthcare, Guangdong, China). In this clinical trial, the World Medical Association's Helsinki Declaration (2000) and related ethical standards are strictly followed, and the institution ethics committee has approved this research program involving human participants.

### 2.2. Materials

All chemicals used were of analytical reagent (AR) grade. Rapamycin (553210-1M) and DL-homocysteine (454-29-5) were purchased from Sigma-Aldrich. Tris HCl, glycine, and other chemicals were also purchased from Sigma-Aldrich. FeTMPyP (133314-07-5) was gained from Cayman Chemical (USA). Antibodies of TFEB (13372-1-AP) for Western blotting were obtained from Proteintech (USA). Antibodies of LC3A/B (Clone D3U4C, 12741) and TFEB (D4L2P, 32361) were purchased from Cell Signaling Technology (USA). Antibodies of p16 (ab51243), p21 (ab09199), and p53 (ab131442) were obtained from Abcam (United Kingdom).

### 2.3. Animal Experiments and Cell Culture

Animal experiments were in line with the Principles of the Use and Care of Animals published by the National Institutes of Health (NIH Publication No. 85-23, Revised 1996) and approved by the Institutional Animal Care and Use Committee of Capital Medical University. Male SD rats (3-month-old, *n* = 15) were randomly divided into two groups: normal diet (control group) or 2.5% methionine diet (HHcy group) for 24 weeks. The serum Hcy levels were detected every two weeks. All rats had free access to water.

MPC-5 (mouse podocyte cells) were incubated in 5% CO_2_ and 95% O_2_ at 37°C with RPMI 1640 DMEM (HyClone, USA, with 10% FBS (Gibco, USA)). When the cells have reached 60-70% confluency, they were treated with Hcy (500 *μ*mol/L) and rapamycin (1 ng/*μ*L). Hcy and FeTMPyP were dissolved in PBS, and rapamycin was diluted to the required concentration with DMSO before treatment. Ad-Atg5 (Atg5 adenovirus) was transduced to the cells at 100 multiplicity of infection (MOI) for 24 h.

### 2.4. Animal Biochemical Analysis

The blood sample was extracted from the abdominal aorta and was centrifuged to collect serum at 4°C and 2500 rpm for 10 min, and stored at -80°C. The serum samples were loaded to the automatic biochemical analyzer (Hitachi 7020, Tokyo, Japan) to detect the levels of tHcy, interleukin-6 (IL-6), and urea.

### 2.5. Morphological Staining

Kidney tissue was fixed with formalin and dehydrated with gradient concentration of ethanol and xylene, embedded in paraffin, and sliced into 4 *μ*m thick sections. Sirius red staining and Masson staining were performed according to the reagent instructions to detect the extent of collagen deposition and fibrosis in kidney tissue. Immunohistochemical staining was used to detect the nitrosation level of the kidney (mouse anti-NT, 1 : 100).

### 2.6. Western Blotting Analysis

Renal tissue protein was extracted with RIPA buffer, PMSF, and protease inhibitors (100 : 1 : 1), homogenized and sonicated on ice for 20 min, and centrifuged at 14000 rpm for 20 minutes at 4°C. Total protein concentration was determined by the Thermo Scientific BCA assay (catalog number). 50 *μ*g of protein was fractionated by 10~12% SDS-PAGE at 80~120 V for 2 h and transferred to the PVDF membrane at 100 V for 1.5 h. Nonspecific binding was covered with 5% skimmed milk powder in Tris-buffered saline containing 0.1% Tween (TBST) for 2 hours at room temperature. Then, the membrane was incubated overnight at 4°C with the appropriate primary antibody (rabbit anti-LC3, 1 : 1000; rabbit anti-TFEB, 1 : 1000; mouse anti-p53, 1 : 1000; rabbit anti-p21, 1 : 1000; rabbit anti-p16, 1 : 1000; and small mouse anti-*β*-actin, 1 : 1000). The membrane was then washed with TBST and incubated with an HRP-conjugated secondary antibody (1: 5000 in TBST) at room temperature for 1 h. Following the washing steps, ECL Plus Substrate (Thermo Scientific, Inc.) was applied to the blot and images were captured in a gel recording system. The relative optical density of the protein bands was analyzed using gel software Image Lab.

### 2.7. Real-Time PCR Analysis

Total RNA was extracted from MPC-5 using TRIzol (t9424, Sigma, USA) following the manufacturer's instructions. The cDNA was generated using HiScript II QRT SuperMix for qPCR (+gDNA wiper) (R223, Vazyme, China) for reverse transcription for 0.5~2 *μ*g of total RNA. Real-time PCR was performed using PowerUp SYBR Master Mix (A25742, Applied Biosystems, USA) on a 7500 Fast Real-time PCR System (Applied Biosystems, USA). GAPDH were used as internal controls for the normalization. All primers used in this study are as follows:

GAPDH-F 5′-TGTGTCCGTCGTGGATCTGA-3′ and GAPDH-R 5′-CCTGCTTCACCACCTTCTTGA-3′, TFEB-F 5′-CAGCAGGTGGTGAAGCAAGAGT-3′ and TFEB-R 5′-TCCAGGTGATGGAACGGAGACT-3′, and LC3-F 5′-CCACCAAGATCCCAGTGATTAT-3′ and LC3-R 5′-TGATTATCTTGATGAGCTCGCT-3′.

### 2.8. Statistical Analysis

Data were analyzed with Prism 6.0 (GraphPad Software Inc., USA). Groups of three or more were analyzed with one-way analysis of variance (ANOVA). Correlations were analyzed using linear correlation analysis. Results are presented as the mean ± standard error of the mean (SEM). A *P* value < 0.05 was considered statistically significant.

## 3. Results

### 3.1. HHcy Led to the Development of Renal Aging

**Figure 1:**
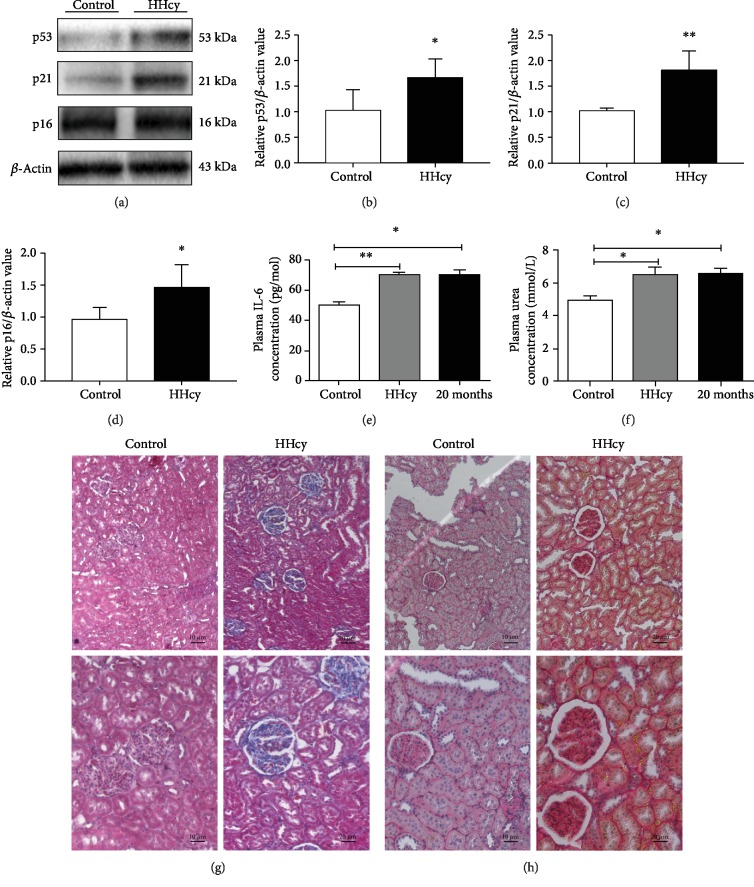
HHcy led to the development of renal aging. (a) Representative Western blot showing the increased expression of aging-related proteins in the kidneys of the HHcy rats (b–d). Quantification of the protein levels of p53, p21, and p16, respectively (^∗^*P* < 0.05, ^∗∗^*P* < 0.01*vs.* Control, *n* = 4-5). (e) Serum level of the aging-related inflammatory cytokine IL-6 (^∗^*P* < 0.05, ^∗∗^*P* < 0.01*vs.* Control, *n* = 6-10). (f) Serum level of urea increased in both HHcy and natural aged mice (^∗^*P* < 0.05*vs.* Control, *n* = 6-10). (g) Masson staining of fibrotic lesions in kidney tissue. (h) Sirius red staining of fibrotic lesions in kidney tissue. Control: control group; HHcy: hyperhomocysteinemia group; IL-6: interleukin-6. Data were expressed as mean ± SEM.

To investigate the relationship between HHcy and renal aging, we analyzed the expression of aging-related proteins and renal function indicators, which reflected the overall aging of the kidneys. The levels of aging-associated proteins p53, p21, and p16 in the kidney were increased, suggesting the occurrence of renal aging in HHcy rats (Figures 1(a)–1(d)). To further determine the aging changes in HHcy rats, we selected 20-month-old natural aging rats as the positive control. In both the natural aging group and the HHcy group, serum levels of the aging-related proinflammatory cytokine IL-6 were elevated (Figure 1(e)). In addition, there was a significant increase in urea level compared with the control group (Figure 1(f)). Further, the histopathological assessment of renal tissue revealed the abnormality of glomerular size and the renal tissue structure in the HHcy group. Masson trichrome staining showed a large amount of interstitial collagen fiber deposition, glomerular atrophy and hardening, renal tubule cystic dilatation, and a large number of focal interstitial fibrosis. Sirius red staining revealed increased fibrosis in the HHcy group (Figures 1(g) and 1(h)).

### 3.2. Autophagic Insufficiency Was Involved in HHcy-Induced Renal Aging

Autophagy has emerged as an important mechanism for ensuring the maintenance and homeostasis need for life-span extension [18, 19].

**Figure 2:**
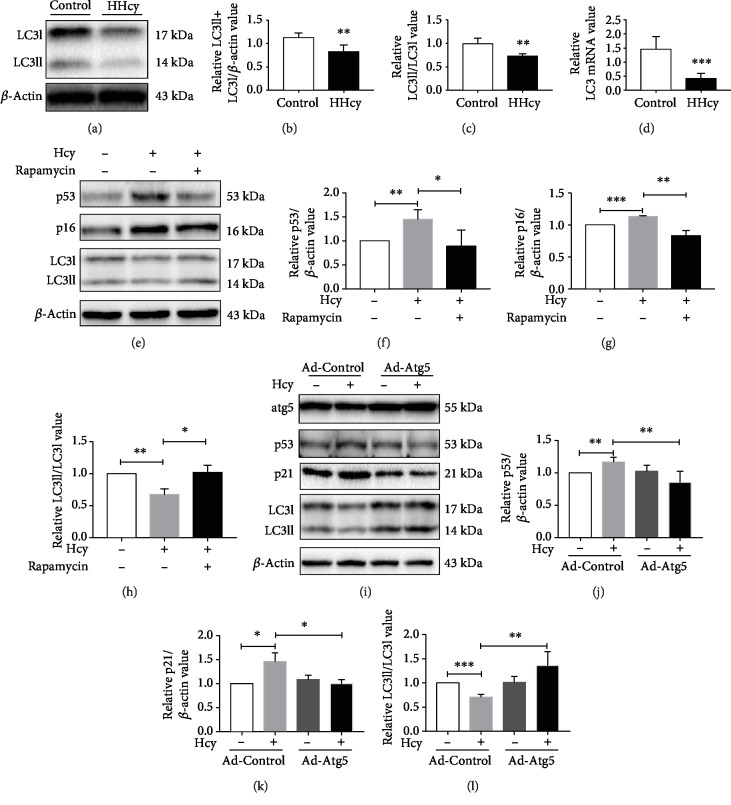
Autophagic insufficiency was involved in HHcy-induced renal aging. (a) Representative Western blot showing the protein expression levels of autophagy in HHcy. Graphs representing (b, c) LC3 by quantitative analysis. (^∗^*P* < 0.05, ^∗∗^*P* < 0.01, *n* = 4-5). (d) Graph representing quantitative analysis showing the mRNA level of LC3 in HHcy (^∗^*P* < 0.05, ^∗∗^*P* < 0.01, and ^∗∗∗^*P* < 0.001; HHcy *vs.* Control; *n* = 5-6). (e) Representative Western blot showing the protein expression levels of aging and autophagy in MPC-5 treated with rapamycin. Graphs representing (f) p53, (g) p16, and (h) LC3 by quantitative analysis (^∗^*P* < 0.05, ^∗∗^*P* < 0.01, and ^∗∗∗^*P* < 0.001, Hcy *vs.* Control, rapamycin *vs.* Hcy, *n* = 3-4). (i) Representative Western blot showing the protein expression levels of aging and autophagy in MPC-5 infected with Atg5 adenovirus. Graphs representing (j) p53, (k) p21, and (l) LC3 by quantitative analysis (^∗^*P* < 0.05, ^∗∗^*P* < 0.01, and ^∗∗∗^*P* < 0.001, Hcy *vs.* Control, Atg5 adenovirus *vs*. Hcy, *n* = 3-4). Control: control group; Hcy: homocysteinemia group; Ad-Atg5: Atg5 adenovirus. Data were expressed as mean ± SEM.

In order to investigate the role of autophagy in HHcy-induced renal aging, we examined the expression of autophagosome-lysosomal-associated marker proteins in kidney tissue. Western blot showed that the expression of the LC3II/LC3I ratio and total LC3 was significantly decreased in HHcy compared with Control, and real-time PCR analysis showed that the mRNA level of LC3 in the HHcy group was decreased significantly. HHcy inhibited not only the production of LC3 from mRNA to protein but also the LC3II/I ratio, thus reducing the occurrence of autophagy, suggesting that HHcy induced a downregulation of autophagy in the kidney (Figures 2(a)–2(d)).

To further verify whether or not autophagy was involved in HHcy-induced renal aging, we used Hcy to stimulate MPC-5 (mouse podocyte cells), in the presence or absence of rapamycin. In view of the fact that in addition to upregulating autophagy, rapamycin itself has a certain alleviation effect on aging, we further infected MPC-5 with Atg5 adenovirus to increase the level of autophagy and examined the expression of senescence markers. The results revealed that HHcy could increase the expression of aging-related proteins of p16, p21, and p53; meanwhile, upregulation of autophagy by rapamycin or Atg5 adenovirus could reverse this phenomenon, which implied that autophagic insufficiency was involved in the process of HHcy-induced renal aging (Figures 2(f)–2(k)).

To further clarify whether or not Hcy can directly cause the aging of MPC-5 by downregulation of autophagy, we also detected the expression of autophagy-lysosome-associated marker proteins. Western blot revealed that the expression of the LC3II/I ratio was increased after rapamycin or Atg5 adenovirus treatment compared with Hcy (Figures 2(h)–2(l)). These results suggested that upregulation of autophagy could rescue the senescence of MPC-5, confirming the essential role of autophagic insufficiency in renal aging induced by HHcy.

### 3.3. HHcy Decreased the Expression of TFEB in Renal Tissue

An important mechanism for ensuring the essential organic metabolism for long-lived life is the transcriptional regulation of autophagy genes. In particular, the helix-loop-helix transcription factor EB (TFEB) controls the expression of many autophagosome-lysosomal-associated genes and plays an important role in prolonging the life-span [20–22].

**Figure 3:**
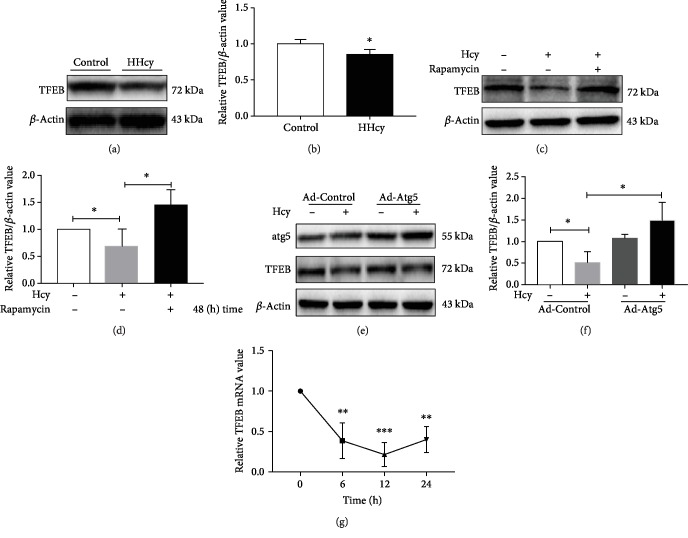
HHcy decreased the expression of TFEB in renal tissue. (a–f) The protein expression of TFEB in kidney extracts of HHcy rats and MPC-5 cells measured by Western blot. (a) Representative Western blot showing the protein expression of TFEB in HHcy. Graphs representing (b) TFEB by quantitative analysis (^∗^*P* < 0.05, HHcy *vs.* Control, *n* = 4-5). (c) Representative Western blot showing the protein expression of TFEB in MPC-5 treated with rapamycin. Graphs representing (d) quantitative analysis (^∗^*P* < 0.05, Hcy *vs.* Control, rapamycin *vs*. Hcy, *n* = 3-4). (e) Representative Western blot showing the protein expression of TFEB in MPC-5 infected with Atg5 adenovirus. Graphs representing (f) (^∗^*P* < 0.05, Hcy *vs.* Control, Atg5 adenovirus *vs.* Hcy, *n* = 3-4). (g) Graphs representing quantitative analysis showing the mRNA level of TFEB in MPC-5 treated with Hcy for 6, 12, and 24 h (^∗^*P* < 0.05, ^∗∗^*P* < 0.01, and ^∗∗∗^*P* < 0.001, Hcy *vs.* Control, *n* = 3-4). Control: control group; Hcy: homocysteinemia group; Ad-Atg5: Atg5 adenovirus. Data were expressed as mean ± SEM.

We showed that HHcy caused a significant decrease in the expression of TFEB in kidney tissue (Figures 3(a) and 3(b)). There was a significant increase in the expression of TFEB in MPC-5 after being treated with rapamycin or Atg5 adenovirus (Figures 3(c)–3(f)). We then monitored the mRNA levels of TFEB at 6 h, 12 h, and 24 h of Hcy challenge. The results showed that 12 h treatment of MPC-5 with Hcy resulted in the lowest level of TFEB mRNA (Figure 3(g)). These results implied that the expression of TFEB, the key regulatory factor of autophagy, was decreased in HHcy-induced renal aging.

### 3.4. Nitrative Stress Participated in Hcy-Induced TFEB Downregulation

**Figure 4:**
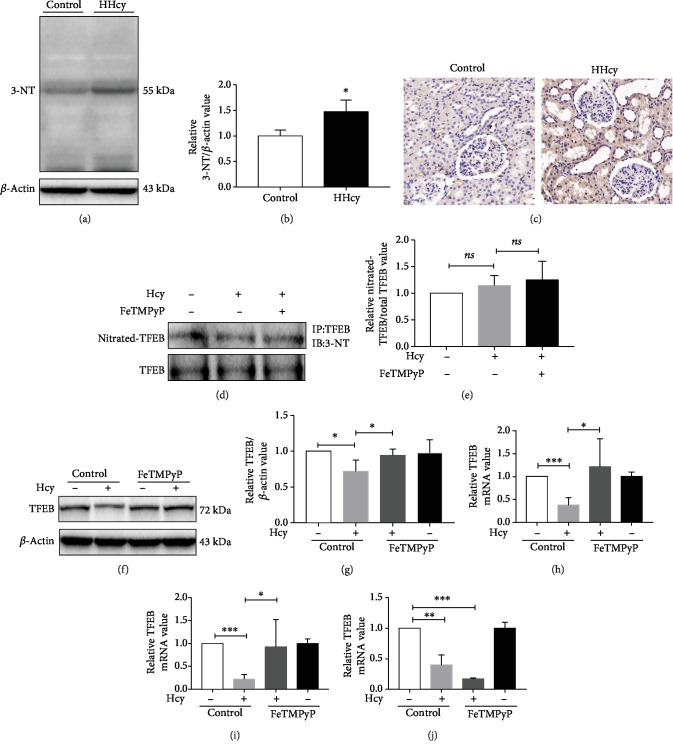
Nitrative stress participated in Hcy-induced TFEB downregulation. (a–c) Representative Western blot and immunohistochemistry detecting 3-NT contents in renal tissue of HHcy rats, respectively. (^∗^*P* < 0.05, HHcy *vs*. Control, *n* = 4-5). (d) Representative immunoprecipitation showing the contents of nitrated TFEB. Graphs representing (e) quantitative analysis (ns, *P* > 0.05, Hcy *vs.* Control, FeTMPyP *vs*. Hcy, *n* = 6). (f) Representative Western blot showing the protein expression of TFEB and 3-NT in MPC-5 treated with FeTMPyP. Graphs representing (g) quantitative analysis (^∗^*P* < 0.05, Hcy *vs.* Control, FeTMPyP *vs.* Hcy, *n* = 3-4). (h–j) Graphs representing quantitative analysis showing the mRNA level of TFEB in MPC-5 treated with FeTMPyP for 6, 12, and 24 h (^∗^*P* < 0.05, ^∗∗^*P* < 0.01, and ^∗∗∗^*P* < 0.001, Hcy *vs.* Control, FeTMPyP *vs.* Hcy, *n* = 3-4). Control: control group; Hcy: homocysteinemia group. Data were expressed as mean ± SEM.

Our previous studies have shown that HHcy increased the level of nitrative stress leading to protein inactivation [23]. To explore the possible relationship between increased nitrative stress and TFEB downregulation, we examined the level of 3-nitrotyrosine (3-NT), the footprint of peroxynitrite (ONOO^−^) in kidney tissue of HHcy rats. The results suggested that HHcy led to an increase in the level of nitrative stress in the renal tissue (Figures 4(a)–4(c)). We then examined the nitrated modification of TFEB after peroxynitrite (ONOO^−^) scavenger FeTMPyP treatment. The immunoprecipitation results showed that Hcy did not nitrate TFEB directly (Figures 4(d) and 4(e)). However, it reduced the expression of TFEB protein. FeTMPyP could partly rescue this phenomenon (Figures 4(f)–4(g)). We measured the mRNA levels of TFEB at 6, 12, and 24 h of Hcy stimulation on MCP-5. The results showed that FeTMPyP rescued TFEB mRNA level at 6 h and 12 h stimulation with Hcy in MPC-5 (Figures 4(h) and 4(i)). The scavenging ability of FeTMPyP disappeared at 24 h (Figure 4(j)). These results indicated that nitrative stress participated in Hcy-induced TFEB downregulation.

### 3.5. Correlation between Renal Function, Autophagy, and Hcy Level in Human

**Figure 5:**
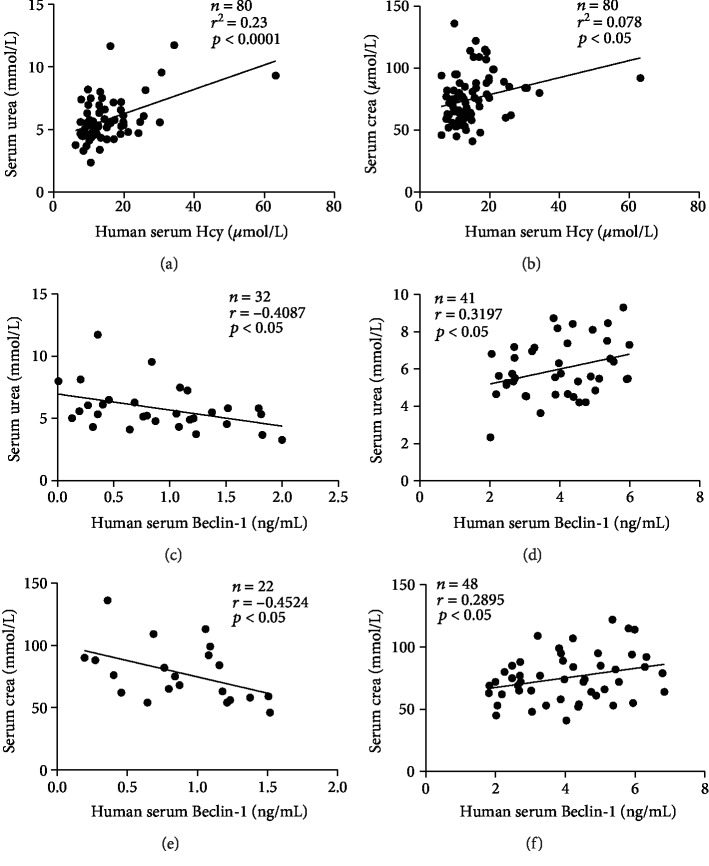
Correlation between renal function, autophagy, and Hcy level in human. (a, b) Correlation between serum urea, creatinine, and Hcy level, respectively (urea: *r*^2^ = 0.23, *P* < 0.0001; creatinine: *r*^2^ = 0.078, *P* < 0.05; *n* = 80). (c) Correlation between urea and Hcy (Beclin-1: 0-2.0 ng/mL, *n* = 32, *r* = −0.4087, *P* < 0.05). (d) Correlation between urea and Hcy (Beclin-1: 2.0-6.0 ng/mL, *n* = 41, *r* = 0.3197, *P* < 0.05). (e) Correlation between creatinine and Hcy (Beclin-1: 0-1.6 ng/mL, *n* = 22, *r* = −0.4524, *P* < 0.05). (f) Correlation between creatinine and Hcy (Beclin-1: 1.6-7.0 ng/mL, *n* = 48, *r* = 0.2895, *P* < 0.05). Crea: creatinine.

Clinical examinations showed a positive correlation between the levels of serum creatinine, urea, and tHcy, respectively, suggesting the association between Hcy and renal injury (Figures 5(a) and 5(b)). Interestingly, when the serum level of the autophagy-related protein Beclin-1 was <2.0 ng/mL, urea was negatively correlated with autophagy, and when Beclin-1 level ≥ 2.0 ng/mL, urea was positively correlated with autophagy (Figures 5(c) and 5(d)). These results implied that patients display lower level of autophagy, and upregulation of autophagy may protect the renal function. However, in the serum, the rise in urea level was accompanied by the increase in Beclin-1, which may lead to aggravation of the kidney damage. The relationship between autophagy and serum creatinine level was similar to urea level. When the serum level of the autophagy-related protein Beclin-1 was <1.6 ng/mL, creatinine was negatively correlated with autophagy, and once Beclin-1 level was ≥1.6 ng/mL, creatinine was positively correlated with autophagy (Figures 5(e) and 5(f)). These results implied that autophagy may be a double-edged sword. Appropriately increasing the autophagy level can exert a protective effect on the renal function; however, excessive autophagy may participate in the damage to the kidney [24].

## 4. Discussion

In this study, we showed four major findings: (1) HHcy led to the development of renal aging. (2) Autophagic insufficiency was involved in HHcy-induced renal aging. (3) HHcy caused a significant decrease in the expression of TFEB, the key regulatory factor of autophagy, in renal tissue. (4) Nitrative stress participated in Hcy-induced TFEB downregulation.

Renal aging is caused by the gradual accumulation of multiple factors [25]. Aging is a biological process characterized by accumulation of damage in the structure and function of cells over time, ultimately leading to death [26]. Regardless of how the precise molecular mechanisms of aging occur, injury at the cellular level can cause a decline in the tissue and organ, including the kidney. Aging of the kidney is related to apparent structural and functional changes. Clinically, renal aging is manifested as decreased urine output and elevated serum creatinine and urea. Morphologically, there are renal cortical atrophy, rough surface, and renal parenchymal ischemic sclerosis [27]. Functionally, there is an imbalance on glomerular capillary wall permeability, renal tubular reabsorption and secretion, acid-base regulation, and disruption of the production of renal hormones and bioactive molecules [28–30].

HHcy is recently regarded as an independent risk or pathogenic factor for the development of renal diseases [31]. HHcy could accelerate ischemia-reperfusion-induced acute kidney injury (AKI) and the following development of renal fibrosis [32]. HHcy emerged in both chronic and end-stage renal diseases. Excessive accumulation of Hcy exacerbates disorders associated with imbalanced homeostasis [6]. The accumulation of Hcy in chronic kidney disease (CKD) could exert toxic effects on the glomerular and tubule interstitial region. In general, elevated serum Hcy is a predictor of accelerated decline in renal function and chronic kidney disease [33]. At present, the research on the relationship between Hcy and renal aging is mostly concentrated in patients with clinically diagnosed primary diseases with renal injury, such as hypertensive renal damage [34] and diabetic nephropathy [35]. Renal insufficiency can lead to accumulation of Hcy, and elevated levels of Hcy can cause a vicious circle of glomerular sclerosis.

In this study, the aging-related proteins in the HHcy group were significantly higher than those in the control group, indicating the occurrence of renal aging at the molecular level. Urea in both HHcy and natural aging rats was greatly increased, showing HHcy-induced impairment of renal function. As markers of renal aging, collagen fiber deposition and fibrosis were also found in the kidney tissue of the HHcy group [36].

The basal level of autophagy arises in many longevity models. Increasing evidences have shown that autophagy is necessary for mediating a long life-span [37]. Abnormal autophagy is considered to be a major factor in aging. In this study, we observed a decreased level of autophagy in the kidney tissue of the HHcy group, which is consistent with the previous findings [38, 39]. We further speculated that the current phenomenon might be caused by the accumulation of Hcy in vivo. Activation of autophagy can protect the kidney's innate cells and reduce the degree of kidney damage in most kidney diseases [40–42]. With key functions of maintaining normal glomerular structure and capillary permeability, podocytes can undergo changes associated with aging [30, 43]. Some researchers found that the weakened autophagy function in podocytes caused the accumulation of oxidized proteins, endoplasmic reticulum stress, and proteinuria, eventually leading to podocyte damage and glomerular sclerosis in a mouse model through knocking out the autophagy-related gene Atg5 [40]. Hcy can directly cause podocyte injury, with subsequent progression of glomerular sclerosis [40, 44]. Our results showed that Hcy could cause the senescence of MPC-5 in vitro. Upregulation of autophagy by rapamycin or Atg5 adenovirus partly inhibited the occurrence of an aging phenotype. These results indicated that autophagy played an important role in Hcy-induced renal aging. The underlying mechanism, however, is still unclear.

The overall autophagy levels are regulated in a dynamic equilibrium at the level of transcriptional regulation to maintain critical cellular functions and ensure adequate energy supply [45]. Numerous studies have shown that TFEB-mediated transcriptional regulation is a key regulatory mechanism of autophagy. TFEB is an important factor regulating the expression of autophagy and lysosomal-associated genes. Under physiological conditions, TFEB is usually localized in the cytosol. Under starvation conditions or when lysosomal function is impaired, TFEB then migrates to the nucleus and transcriptionally regulates the expression of downstream genes [20]. TFEB is positively correlated with the occurrence of autophagy [46, 47]. In this study, we found that expression of TFEB protein was reduced in HHcy. This is consistent with previous reports and further supports the notion that TFEB is a bridge between autophagy and aging [48, 49]. In addition, our study showed for the first time that TFEB was downregulated in both the kidney of HHcy rats and MPC-5 treated with Hcy.

As an important posttranslational modification of proteins, nitrative stress refers to the nitration of aromatic amino acid residues. Reactive oxygen species (ROS) rapidly reacts with nitric oxide (NO) to produce a large number of ONOO^−^, causing nitration of aromatic amino acid residues such as tyrosine. This modification usually induces a pathological state in which functions of proteins/enzyme are impaired, resulting in toxic effects on the cells. A variety of pathological factors such as age, high glucose, and HHcy can promote the increase in nitrative stress in the body. HHcy stimulates the production of NO and ROS, producing a large amount of ONOO^−^, therefore increasing the nitrative stress sharply [23].

Our previous study has found that pretreatment with the ONOO^−^ scavenger FeTMPyP can inhibit nitrative stress and increase autophagy [50]. As a transcription factor, the transcriptional activity of TFEB can be regulated by both posttranslational modification and interaction of protein-protein. To explore the possible relationship between TFEB and nitration, we used Hcy to stimulate MPC-5, in the presence or absence of FeTMPyP. It was found that FeTMPyP increased the level of TFEB protein and mRNA in MPC-5 cells, indicating that Hcy was most likely to reduce the expression of TFEB by elevation of nitrative stress, thereby decreasing autophagy and ultimately promoting aging. We speculate that nitration by HHcy plays an important role in the inactivation of TFEB. Inactivation of TFEB mainly could embody two aspects: Firstly, TFEB itself would undergo nitration, which directly affects the expression of genes related to the autophagy-lysosome pathway. Secondly, nitration of factors in the upstream of TFEB will downregulate the expression of TFEB, thus affecting the expression of genes related to the autophagy-lysosome pathway. Based on the present results, we assume that the latter is more likely to exist. Through searching the PROMO website, we have predicted that C/EBP*β* is the transcription factor of TFEB and speculated whether or not C/EBP*β* would undergo nitration, thus downregulating the expression of TFEB and ultimately affecting the transcription of genes related to the autophagy-lysosome pathway. Of course, this is only a hypothesis at present and a large amount of experimental work would be needed to identify it in the future.

In clinical trials, the level of serum tHcy in patients with HHcy was positively correlated with serum urea and creatinine levels, further validating that HHcy can promote the development of kidney damage. Recently, evidence that autophagy and autophagic factors are intimately intertwined at many levels with secretion in eukaryotic cells has emerged [51]. Secretory protein markers of autophagy are a form of unconventional protein secretion [52]. Kraya et al. have proposed an autophagosome-dependent secretion mechanism for protein markers of autophagy [53]. Beclin-1, as a positive regulator of autophagy, plays an important role in the occurrence and development of autophagy. It is reported that autophagy biomarkers Beclin-1 and LC3B can be detected by the enzyme-linked immunosorbent assay (ELISA) in serum and cerebrospinal fluid (CSF) [54]. Based on these work, the difference of Beclin-1 levels in serum of patients in our study is coming from the different levels of autophagy to some degree. Interestingly, at the basal level of autophagy, the levels of serum urea and creatinine were normal. However, the rise of creatinine and urea levels was accompanied by the increase in Beclin-1 expression, which may lead to the aggravation of kidney damage. This phenomenon may be due to the dual effects of autophagy. Similar to previous reports, our findings confirmed that appropriately increasing the autophagy level can exert a protective effect; however, excessive autophagy may be harmful.

In summary, this study showed that HHcy promoted the development of renal aging, accompanied by the downregulation of autophagy and the occurrence of nitrative stress. Further, we demonstrated that nitrative stress led to decreased expression of TFEB, the key regulatory factor of autophagy, implying that nitrative stress-related autophagic insufficiency participates in HHcy-induced renal aging. This study would provide a novel insight into the mechanism and therapeutic strategy for renal aging.

## Data Availability

The data used to support the findings of this study are available from the corresponding authors upon request.
